# High density lipoproteins, disease severity and clinical outcomes in patients with idiopathic pulmonary fibrosis

**DOI:** 10.1186/s12931-026-03511-x

**Published:** 2026-02-17

**Authors:** Anna J. Podolanczuk, Hillary Mulder, John S. Kim, Megan L. Neely, Robert J. Kaner, Jamie L. Todd

**Affiliations:** 1 Weill Cornell Medicine, New York, USA; 2 Duke Clinical Research Institute, Durham, USA; 3 University of Virginia, Charlottesville, USA; 4 Duke University Medical Center, Durham, USA

**Keywords:** Lipids, Biomarkers, Disease progression, Lung diseases, interstitial

## Abstract

**Background:**

Dysregulation of lipid metabolism is implicated in the pathogenesis of idiopathic pulmonary fibrosis (IPF). We assessed associations between circulating levels of high-density lipoprotein-cholesterol (HDL-C) and its components apolipoprotein A-1 (ApoA1) and paraoxonase-1 (PON-1) and disease severity and outcomes in patients with IPF.

**Methods:**

The cohort comprised 284 patients enrolled in the IPF-PRO Registry. We analyzed associations between HDL-C, ApoA1 and PON-1 levels at enrollment and measures of disease severity at enrollment (using linear regression) and outcomes during follow-up (using Cox models). Models were adjusted for demographic and clinical variables, including cardiovascular disease and statin use, assessed at enrollment.

**Results:**

In unadjusted models, higher ApoA1 and lower PON-1 were associated with higher FVC % predicted at enrollment. Median follow-up was 40 months. In unadjusted models, higher ApoA1 at enrollment was associated with lower risks of all-cause mortality (hazard ratio [HR] 0.32 [95% CI: 0.16, 0.64] per unit higher log_2_ ApoA1) and respiratory hospitalization (HR 0.25 [0.11, 0.57] per unit higher log_2_ ApoA1). In analyses adjusted for demographic and clinical variables, the association with respiratory hospitalization remained significant (HR 0.30 [95% CI: 0.10, 0.88; *p* = 0.029]), but the association with all-cause mortality was no longer significant (HR 0.44 [95% CI: 0.18, 1.08; *p* = 0.074]). Levels of PON-1 or HDL-C were not associated with all-cause mortality or respiratory hospitalization.

**Conclusion:**

In a real-world cohort of patients with IPF, a higher circulating level of ApoA1 was associated with a lower risk of respiratory hospitalization. ApoA1 is a potential prognostic biomarker in patients with IPF.

## Introduction

Idiopathic pulmonary fibrosis (IPF) is a progressive fibrosing interstitial lung disease (ILD) characterized by decline in lung function and high mortality [1]. While all patients with IPF experience disease progression, the rate and pattern of decline are highly variable [2]. This clinical heterogeneity presents significant challenges for patient management and therapeutic decision making. Biomarkers that predict clinically relevant outcomes in patients with IPF are needed to improve prognostication and advance our understanding of the mechanisms underlying progressive lung fibrosis.

Dysregulation of cellular metabolic pathways, including lipid metabolism, has been implicated in the pathogenesis of IPF [3, 4]. High-density lipoprotein cholesterol (HDL-C) is a blood biomarker that modulates lipid metabolism and vascular inflammation. Patients with IPF have lower levels of HDL-C than healthy individuals [5]. Apolipoprotein A-1 (ApoA1) and paraoxonase 1 (PON-1) are key components of HDL-C that have anti-inflammatory and antioxidant properties [4, 6, 7]. ApoA1 promotes cholesterol efflux from endothelial and immune cells, thereby reducing cellular cholesterol and inflammation, while PON-1 protects from oxidative modification, preserving vascular and immune homeostasis. These mechanisms have been implicated in cardiovascular disease and could also play a role in lung fibrosis. ApoA1 levels in bronchoalveolar lavage fluid have been shown to be lower in patients with IPF than in individuals without IPF [8]. Treatment with ApoA1 attenuated bleomycin-induced lung injury in mice [8] and, in transgenic mouse models, ApoA1 overexpression attenuated silica-induced alveolar fibrotic nodules [9]. Circulating PON-1 levels have also been shown to be lower in patients with IPF than in healthy individuals [5, 10]. These studies suggest that HDL-C and its key components ApoA1 and PON-1 may exert protective effects in the pathogenesis of lung fibrosis.

In order to evaluate the potential of HDL-C and its associated proteins as biomarkers and/or therapeutic targets in pulmonary fibrosis, we used data from the IPF-PRO Registry to assess associations between circulating levels of HDL-C, ApoA1 and PON-1 and disease severity and outcomes in patients with IPF.

## Methods

Patients with IPF that was diagnosed or confirmed at the enrolling center in the past 6 months were enrolled into the IPF-PRO Registry (NCT01915511) [11] at 46 sites across the US. Blood samples and clinical data were collected at enrollment. Clinical data were collected as part of patients’ routine care until death, lung transplant, or withdrawal from the registry. The cohort for these analyses was drawn from 300 patients being followed in the registry as of 1 February 2017, who had data on height, sex, age, forced expiratory volume in 1 s (FEV_1_), forced vital capacity (FVC) and diffusing capacity of the lungs for carbon monoxide (DLco) (i.e. data on key clinical variables) at enrollment, as well as biosamples. Plasma ApoA1 and PON-1 levels were measured using an aptamer-based platform (SOMAscan, SOMALogic, Inc) in all 300 participants. Participants with sufficient remaining plasma for HDL-C level measurement (*n* = 284) were included in the analysis. HDL-C was measured using a standard clinical assay at a single laboratory (Duke Molecular Physiology Institute). Values were log_2_ transformed before analysis.

Baseline characteristics of the cohort are presented as median (25th, 75th percentiles) for continuous measures and n (%) for categorical measures. Linear regression models were used to assess associations between HDL-C, ApoA1 and PON-1 levels and measures of disease severity (FVC % predicted, DLco % predicted, composite physiologic index [CPI] [12], and gender-age-physiology [GAP] index) [13] at enrollment. Cox proportional hazards regression models were used to assess associations between HDL-C, ApoA1 and PON-1 levels at enrollment and time to the following outcomes: all-cause mortality; respiratory hospitalization (determined by the investigator at the enrolling center); relative decline in FVC (L) > 10%. All models were unadjusted and adjusted for demographic and clinical variables at enrollment: age, sex, race (white vs. non-white), smoking status, body mass index (BMI), C-reactive protein, triglycerides, low-density lipoprotein cholesterol, coronary artery disease, diabetes, heart failure, and use of statins, antifibrotic drugs, and oral corticosteroids. FVC (L) at enrollment was included as an additional covariate for the time-to-event outcomes. Continuous covariates were included as natural cubic splines to account for potential non-linear relationships. An interaction term was included in both the linear regression and Cox models to understand if the impact of HDL-C, ApoA1, or PON-1 level, respectively, on outcomes was different in patients on vs. not on a statin at enrollment. The linearity assumption for HDL-C, ApoA1 and PON-1 with respect to outcomes was assessed using a lack-of-fit test that compared a natural cubic spline fit to a linear fit. The proportional hazards assumption for HDL-C, ApoA1 and PON-1 with respect to outcomes was evaluated using weighted Schoenfeld residuals.

Analyses were performed by the Duke Clinical Research Institute using SAS version 9.4 (SAS Institute, Cary, NC) and R version 3.6.1. or higher (R Core Team).

## Results

At enrollment, the median age of the cohort was 70.0 years; 75.0% were male; 46.8% were taking antifibrotic drugs and 52.8% were taking statins (Table 1). Median (Q1, Q3) FVC was 70.0 (61.2, 80.2) % predicted, DLco was 40.8 (31.8, 49.5) % predicted and CPI was 53.3 (46.2, 60.4).

**Table 1:** Patient characteristics at enrollment (*n* = 284)

Age, years	70.0 (65.0, 75.0)
Male	213 (75.0)
White	266 (93.7)
Smoking status	
Current	2 (0.7)
Past	191 (67.3)
Never	91 (32.0)
Body mass index, kg/m^2^	29.4 (26.3, 32.8)
FVC % predicted	70.0 (61.2, 80.2)
DLco % predicted	40.8 (31.8, 49.5)
Composite physiologic index	53.3 (46.2, 60.4)
GAP Index	3 (4, 5)
GAP Stage	
I (GAP Index 0–3)	76 (26.8)
II (GAP Index 4–5)	168 (59.2)
III (GAP Index 6–8)	40 (14.1)
Coronary artery disease	86 (30.3)
Diabetes	56 (19.7)
Heart failure	21 (7.5)
Supplemental oxygen use*	
At rest	56 (19.8)
With activity	44 (15.5)
None	183 (64.7)
Antifibrotic drug use	133 (46.8)
Pirfenidone	92 (32.4)
Nintedanib	41 (14.4)
Statin use	150 (52.8)
Oral corticosteroid use	36 (12.7)
C-reactive protein, mg/dL	4.1 (1.7, 8.9)
Triglycerides, mg/dL	134.0 (98.0, 190.5)
Low-density lipoprotein cholesterol, mg/dL	94.7 (77.1, 119.7)
High-density lipoprotein cholesterol, mg/dL	48.9 (40.4, 61.0)
ApoA1, RFU	15,284 (13377, 16811)
PON-1, RFU	214 (193, 244)

Data are median (Q1, Q3) or n (%). *n=283 with available information. *ApoA1* apolipoprotein A-1, *DLco* diffusing capacity of the lungs for carbon monoxide, *FVC* forced vital capacity, *PON-1* paraoxonase 1, *RFU* relative fluorescence unit

**Fig. 1:**
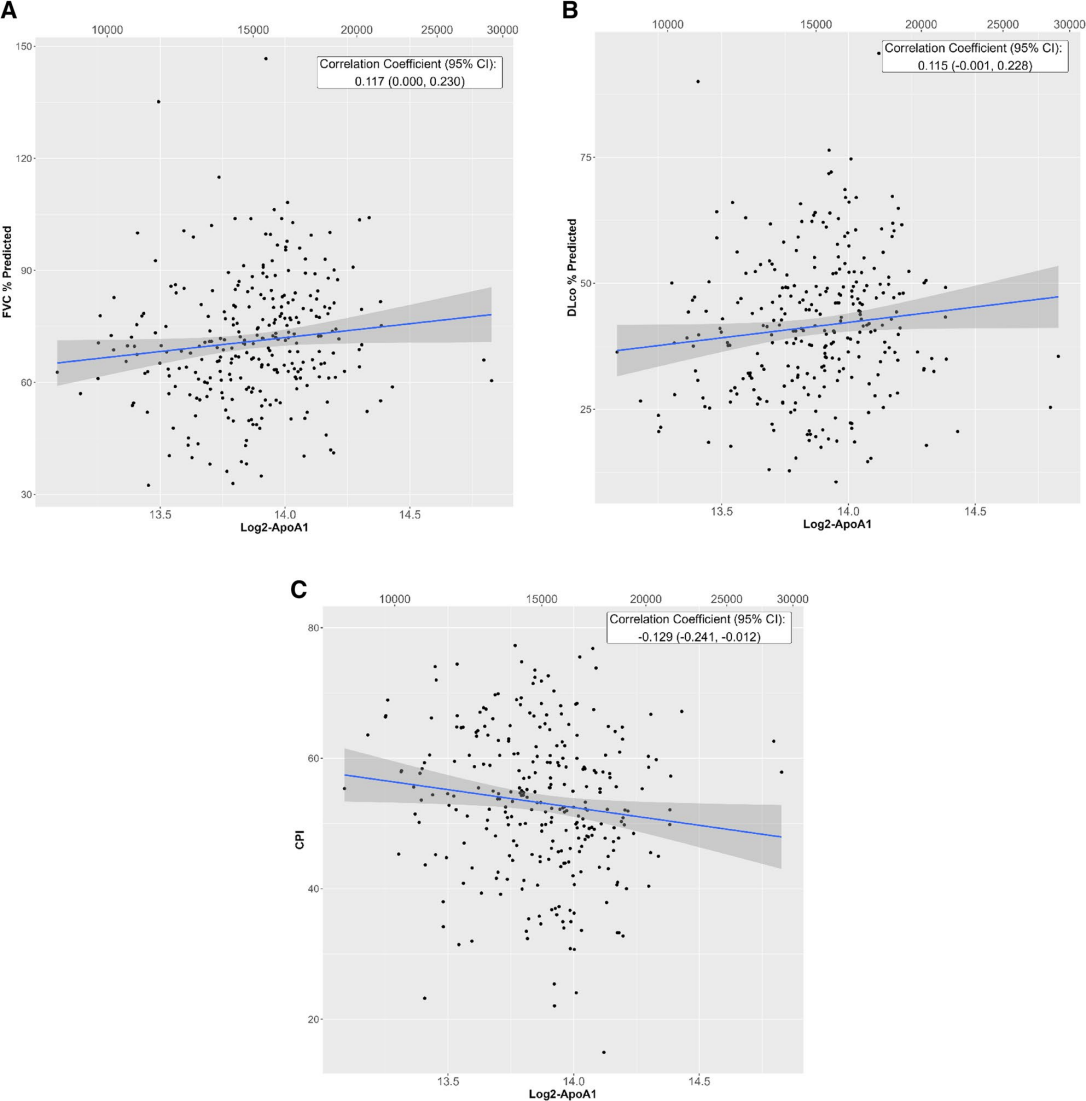
Correlations between ApoA1 and measures of IPF severity at enrollment. (**A**) FVC % predicted. (**B**) DLco % predicted. (**C**) CPI. ApoA1: apolipoprotein A-1; CPI: composite physiologic index; DLco: diffusing capacity of the lungs for carbon monoxide; FVC, forced vital capacity; IPF: idiopathic pulmonary fibrosis. The bottom axis displays the log-transformed ApoA1 values, and the top axis displays the corresponding ApoA1 values on the original scale

In unadjusted models, a higher level of ApoA1 was significantly associated with higher FVC % predicted, lower CPI, and lower GAP index (but not with a difference in DLco % predicted) at enrollment (Table 2; Fig. 1). Per unit higher log_2_ concentration of ApoA1, the difference in FVC % predicted was 7.4 (95% CI: 0.0, 14.9; *p* = 0.049), the difference in CPI was − 5.5 (95% CI: -10.4, -0.5; *p* = 0.030) and the difference in GAP index was − 0.7 (95% CI -1.3, -0.1; *p* = 0.020). These effect estimates were similar after adjustment for demographic and clinical variables but were no longer statistically significant (Table 2). A higher level of PON-1 was significantly associated with lower FVC % predicted at enrollment in unadjusted and adjusted models, but not with DLco % predicted, CPI, or GAP index (Table 2). Levels of HDL-C were not significantly associated with any disease severity measure at enrollment. There were no significant interactions with statin use at enrollment.

**Table 2:** Associations between ApoA1, PON-1 and HDL-C and measures of IPF severity at enrollment

	Unadjusted analyses		Adjusted analyses*	
	Difference in disease severity measure per unit higher log_2_ concentration (95% CI)	*p*-value	Difference in disease severity measure per unit higher log_2_ concentration (95% CI)	*p*-value
**FVC % predicted**				
ApoA1	7.4 (0.0, 14.9)	0.049	6.9 (− 2.7, 16.5)	0.156
PON-1	-6.9 (-11.9, -1.8)	0.008	-6.5 (-11.5, -1.5)	0.012
HDL-C	3.4 (− 1.3, 8.0)	0.152	2.3 (− 3.7, 8.3)	0.453
**DLco % predicted**				
ApoA-1	6.1 (-0.1, 12.3)	0.053	4.3 (-3.5, 12.0)	0.277
PON-1	-1.3 (-5.6, 3.0)	0.556	-1.1 (-5.2, 3.0)	0.592
HDL-C	-2.1 (-6.0, 1.8)	0.288	0.7 (-4.2, 5.5)	0.789
**CPI**				
ApoA1	-5.5 (-10.4, -0.5)	0.030	-4.5 (-10.8, 1.8)	0.159
PON-1	2.1 (-1.3, 5.5)	0.233	2.1 (-1.3, 5.4)	0.226
HDL-C	0.4 (-2.7, 3.5)	0.782	-1.7 (-5.6, 2.2)	0.398
**GAP Index**				
ApoA1	-0.7 (-1.3, -0.1)	0.020	-0.3 (-1.0, 0.3)	0.345
PON-1	-0.1 (-0.4, 0.3)	0.748	0.1 (-0.3, 0.5)	0.709
HDL-C	0.3 (-0.1, 0.7)	0.153	0.4 (0.1, 0.7)	0.026

*Adjusted for age, sex, race (white vs non-white), smoking status, body mass index, C-reactive protein, triglycerides, low-density lipoprotein cholesterol, coronary artery disease, diabetes, heart failure, and use of statins, antifibrotic drugs, and oral corticosteroids at enrollment. Continuous adjustment variables were included as natural cubic splines to account for any potential non-linear relationships. *ApoA1* apolipoprotein A-1, *CI* confidence interval, *CPI* composite physiologic index, *DLco* diffusing capacity of the lungs for carbon monoxide, *FVC* forced vital capacity, *HDL-C* high-density lipoprotein C, *IPF* idiopathic pulmonary fibrosis, *PON-1* paraoxonase-1. All *p*-values for interaction test >0.05

**Fig. 2:**
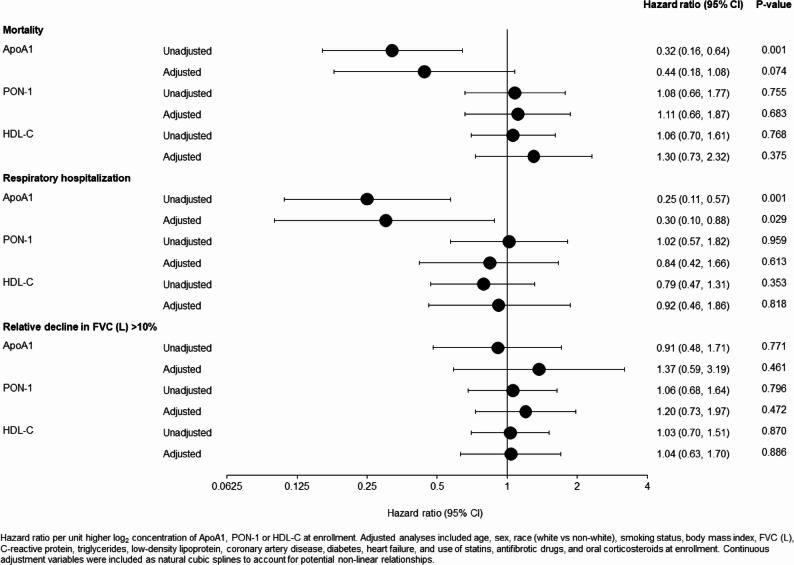
Associations between ApoA-1, PON-1 and HDL-C at enrollment and time to outcomes. ApoA1: apolipoprotein A-1; CI: confidence interval; FVC: forced vital capacity; HDL-C: high-density lipoprotein C; PON-1: paraoxonase-1

Over a median (Q1, Q3) follow-up of 40 (20.4, 59.1) months, 89 (31.3%) patients had a respiratory hospitalization, 128 (45.1%) died from any cause, and 155 (54.6%) had a relative decline in FVC (L) > 10%. In unadjusted models, ApoA1 at enrollment was associated with a significantly lower risk of respiratory hospitalization (hazard ratio 0.25 [95% CI: 0.11, 0.57] per unit higher log_2_ ApoA1; *p* = 0.001]) (Fig. 2). This association remained significant after adjustment for demographic and clinical factors (adjusted hazard ratio 0.30 [95% CI: 0.10, 0.88; *p* = 0.029]). ApoA1 was also associated with lower risk of all-cause mortality in unadjusted analyses (hazard ratio 0.32 [95% CI: 0.16, 0.64] per unit higher log_2_ ApoA1; *p* = 0.001]) (Fig. 2). However, the association with mortality was not statistically significant after adjustment (adjusted hazard ratio 0.44 [95% CI: 0.18, 1.08; *p* = 0.074]) (Fig. 2). Levels of PON-1 or HDL-C at enrollment were not significantly associated with any of the outcomes assessed (Fig. 2). Effects were similar in those on vs. not on statin, as well as on antifibrotic therapy (nintedanib or pirfenidone) vs. none at enrollment (*p*-values for interaction tests with statin use and nintedanib/pirfenidone use at enrollment all > 0.05).

## Discussion

In this analysis of data from the multicenter prospective IPF-PRO Registry, a higher circulating level of ApoA1 at enrollment was associated with less severe disease at enrollment and a lower risk of mortality and respiratory hospitalization during follow-up in unadjusted analyses. The association between ApoA1 and the risk of respiratory hospitalization remained significant after adjusting for cardiovascular and metabolic comorbidities, inflammatory status (C-reactive protein), and use of statins. Adjusting for these variables may have contributed to the loss of the statistically significant association between ApoA1 and mortality risk in our cohort. The lack of association between ApoA1 and relative FVC decline is consistent with prior IPF biomarker studies. This may be because FVC decline can occur gradually and is influenced by measurement variability, patient effort, and comorbidities, making it difficult to correlate with blood biomarkers across a broad patient population. In contrast, blood-based biomarkers that predict mortality more often reflect systemic disease burden or end-stage fibrotic processes, which are more uniform across patients.

Our findings align with prior studies and support a protective role for ApoA1 in lung fibrosis. In a retrospective analysis of 371 patients with IPF, lower ApoA1 levels (< 140 mg/dL) were associated with a higher risk of death over a median follow-up of 18 months, independent of clinical factors associated with mortality in the cohort (smoking history, BMI, GAP score, antifibrotic drug use) [14]. Oh et al. reported a significant correlation between lower ApoA1 and greater disease severity at baseline based on DLco and GAP score [14]. In a separate study of 28 patients, lower ApoA1 level was associated with higher fibrosis score on HRCT [8]. These findings support further investigation of ApoA1 as a prognostic biomarker. However, moving it toward clinical use will require defining optimal cut-offs that predict risk across diverse cohorts, integrating it into existing risk-prediction models such as the GAP score [13] to assess its added value, correlating its longitudinal trajectory with other markers of disease progression and evaluating its performance in prospective studies.

The mechanisms by which ApoA1 may affect progression of pulmonary fibrosis have not been established, but multiple studies implicate dysregulated metabolic pathways as contributors to disease pathogenesis. Metabolic reprogramming of lung fibroblasts and alveolar epithelial cells occurs in IPF and blood biomarkers of cellular metabolic derangements, including increased mitochondrial DNA, sirtuins and low thyroid hormone, have been shown to be associated with disease severity and progression [15–18]. ApoA1 may influence cellular metabolism through its role in mediating reverse cholesterol transport, reducing cholesterol accumulation in alveolar macrophages and other cells, thereby regulating lung lipid homeostasis [4]. Similar to its protective effect in systemic cardiovascular disease, ApoA1 may exert beneficial effects on pulmonary vascular inflammation and remodeling, processes that contribute to the progression of IPF [19]. ApoA1 has been shown to directly inhibit transforming growth factor (TGF)-b1-induced epithelial-to-mesenchymal transition in alveolar epithelial cells, and treatment with ApoA1 attenuated bleomycin-induced lung injury in animal models of lung fibrosis [8, 20].

We also observed that a higher level of PON-1 was associated with lower FVC % predicted at enrollment. This finding was somewhat surprising given that PON-1 is known to have anti-inflammatory and anti-oxidant properties [7, 21] and that its overexpression in experimental models of lung injury is associated with reduced lung inflammation and remodeling [22]. HDL-associated PON-1 hydrolyzes oxidized LDL-cholesterol, thereby decreasing lipid peroxide accumulation [21]. It is important to note that PON-1 protein abundance measured by the SOMAscan platform does not capture its catalytic function in vivo. It is possible that PON-1 is present but its activity is impaired in patients with IPF, especially in the setting of oxidative stress or inflammation. Assays that directly measure PON-1 enzymatic activity may provide a more accurate assessment of its biological role and relevance in IPF.

In our analyses, we found no associations between HDL-C levels and disease severity or outcomes. Prior studies have shown inconsistent associations between HDL-C and mortality in patients with IPF. Oh et al. reported that HDL-C levels were not significantly associated with the risk of death [14]. However, a previous analysis of 266 patients with IPF found that higher levels of small HDL particles were associated with less severe disease based on GAP score, and with a lower risk of death or lung transplant over 1, 2, or 3 years [23]. A study of 123 patients found that those with lower levels of HDL-C had a greater risk of death over 4 years [5]. These inconsistent results reflect the heterogeneity of HDL-C particles. Small dense HDL particles are protein-rich and lipid-poor, and exhibit enhanced atheroprotective effects due to their high capacity for cholesterol efflux [24]. Their levels may be more biologically relevant than total HDL-C, potentially explaining why ApoA1, a key structural component of HDL-C and mediator of its function, performed better prognostically than total HDL-C levels.

Strengths of our analyses include the use of a large multicenter cohort of patients with IPF and the prospective recording of hospitalizations. We accounted for a number of potential confounders, including other cardiovascular disease risk factors, coronary artery disease and medications. Limitations of our analyses include that the cohort was enrolled at expert centers in the US and may not be representative of the general population of patients with IPF. While the IPF-PRO Registry enrolled patients with newly diagnosed or confirmed IPF, there was heterogeneity in disease stage and time from symptom onset, reflecting the challenges patients face in achieving timely diagnosis and care. We do not have data on diet, exercise and alcohol consumption, which could cause residual confounding. We adjusted for baseline comorbidities and medication use, but recognize that the development of new comorbidities and changes in therapy, including statins and antifibrotic drugs, during follow-up may have influenced the outcomes and are a source of residual confounding. We do not have data on additional endpoints such as initiation of oxygen therapy and acute exacerbations of IPF.

In conclusion, among individuals with IPF in a real-world cohort, a higher circulating level of ApoA1 was associated with better lung function at enrollment and a lower risk of respiratory hospitalization during follow-up. These findings highlight the potential importance of metabolic pathways involved in lipid homeostasis in IPF pathogenesis and support further investigation of the roles of ApoA1 and HDL-C in the progression of pulmonary fibrosis. Future studies should evaluate whether some individuals with IPF might benefit from therapies targeting ApoA1 and/or cholesterol homeostasis, and whether the measurement of these biomarkers provides additional prognostic information in clinical practice.

## Data Availability

The datasets analyzed during the current study are not publicly available, but are available from the corresponding author on reasonable request.

## Abbreviations

ApoA1: Apolipoprotein A-1
BMI: Body mass index
CPI: Composite physiologic index
DLco: Diffusing capacity of the lungs for carbon monoxide
FEV_1_: Forced expiratory volume in 1 second
FVC: Forced vital capacity
GAP: Gender, age and physiology
HDL-C: High-density lipoprotein cholesterol
ILD: Interstitial lung disease
IPF: Idiopathic pulmonary fibrosis
PON-1: Paraoxonase 1
